# Modulation of Matrix Metalloproteinases Activity in the Ventral Horn of the Spinal Cord Re-stores Neuroglial Synaptic Homeostasis and Neurotrophic Support following Peripheral Nerve Injury

**DOI:** 10.1371/journal.pone.0152750

**Published:** 2016-03-30

**Authors:** Giovanni Cirillo, Anna Maria Colangelo, Ciro De Luca, Leonilde Savarese, Maria Rosaria Barillari, Lilia Alberghina, Michele Papa

**Affiliations:** 1 Laboratory of Neuronal Networks, Department. of Mental and Physical Health and Preventive Medicine, Second University of Naples, 80138 Naples, Italy; 2 Laboratory of Neuroscience “R. Levi-Montalcini”, Department of Biotechnology and Biosciences, University of Milano-Bicocca, Milano, Italy; 3 SYSBIO, Centre of Systems Biology, University of Milano-Bicocca, Milano, Italy; Toronto University, CANADA

## Abstract

Modulation of extracellular matrix (ECM) remodeling after peripheral nerve injury (PNI) could represent a valid therapeutic strategy to prevent maladaptive synaptic plasticity in central nervous system (CNS). Inhibition of matrix metalloproteinases (MMPs) and maintaining a neurotrophic support could represent two approaches to prevent or reduce the maladaptive plastic changes in the ventral horn of spinal cord following PNI. The purpose of our study was to analyze changes in the ventral horn produced by gliopathy determined by the suffering of motor neurons following spared nerve injury (SNI) of the sciatic nerve and how the intrathecal (i.t.) administration of GM6001 (a MMPs inhibitor) or the NGF mimetic peptide BB14 modulate these events. Immunohistochemical analysis of spinal cord sections revealed that motor neuron disease following SNI was associated with increased microglial (Iba1) and astrocytic (GFAP) response in the ventral horn of the spinal cord, indicative of reactive gliosis. These changes were paralleled by decreased glial aminoacid transporters (glutamate GLT1 and glycine GlyT1), increased levels of the neuronal glutamate transporter EAAC1, and a net increase of the Glutamate/GABA ratio, as measured by HPLC analysis. These molecular changes correlated to a significant reduction of mature NGF levels in the ventral horn. Continuous i.t. infusion of both GM6001 and BB14 reduced reactive astrogliosis, recovered the expression of neuronal and glial transporters, lowering the Glutamate/GABA ratio. Inhibition of MMPs by GM6001 significantly increased mature NGF levels, but it was absolutely ineffective in modifying the reactivity of microglia cells. Therefore, MMPs inhibition, although supplies neurotrophic support to ECM components and restores neuro-glial transporters expression, differently modulates astrocytic and microglial response after PNI.

## Introduction

Matrix metalloproteinases (MMPs) are a family of enzymes essential for the plastic response of the extracellular matrix (ECM) in the central nervous system (CNS) [1]. The notion of tripartite synapse, consisting of pre- and post-synaptic neurons and glial cells, has progressively evolved into the concept of tetrapartite synapse, in which the ECM is included [2]. Through the formation of mature ECM perineuronal nets (PNNs), ECM ensures the synaptogenesis and synaptic maturation, reshaping of neuronal connections [3] but also angiogenesis, and vascular integrity [4]. This system, moreover, has strong connections with cytokines and neurotrophins, such as nerve growth factor (NGF) [5].

Activation of MMPs allows cell migration, blood–brain barrier disruption, cytokines production and release of a number of inflammatory and neurodegenerative disorders [6–7]. Accordingly, in experimental models of peripheral nerve and spinal cord injury or neurodegenerative disorders, modulation of MMPs activity, through administration of specific MMPs inhibitors, was found to restore neuronal plasticity [8–9] and boost functional recovery [10].

MMPs are expressed at very low levels in the adult CNS, secreted as inactive pro-enzymes (pro-MMPs) by several cell types, including macrophages, neurons and glial cells [11] and activated by a selective and finely regulated cleavage. Pro-MMP-9/MMP-9 regulates neurotrophins activity and degradation by the tissue plasminogen activator (tPA)/plasminogen/plasmin system [12–13]: plasmin promotes the activating-cleavage of MMP-9, which rapidly degrades NGF protein; in turn, NGF increase up-regulates MMP-9 expression through the neurotrophin TrkA receptor in a fine-regulated feedback loop [14–15]. Pro-MMPs (-2 and -9) were found activated in pyramidal cells of the motor cortex and spinal astrocytes in amyotrophic lateral sclerosis (ALS) patients [16], suggesting an alteration of the structural integrity of the ECM in ALS [17]. MMP-2 and MMP-9 are also increased in Alzheimer’s disease (AD) patients [18] and in neural progenitor cells (NPC) of subventricular and subgranular zone of the dentate gyrus in brain ischemic animal models [19]. Our group, moreover, reported that intrathecal (i.t.) infusion of GM6001, a MMPs inhibitor, increased the endogenous NGF content, and restored synaptic homeostasis in the dorsal horn of spinal cord after peripheral nerve injury (PNI) [13].

The spared nerve injury (SNI) of the sciatic nerve is currently considered a model to induce persistent neuropathic pain [20] and associated to an intense glial reaction in the dorsal horn of the spinal cord that has been demonstrated to correlate with mechanisms of neuropathic pain [13]. However, following a peripheral axotomy or axonal crush, also spinal motor neurons are denervated and this axonal insult determines changes of neuroglial homeostasis also in the ventral horn [21]. Moreover, the peripheral insult gives the opportunity to perturb the spinal circuitry and study the motor neurons environment and behavior “from the periphery”. This could represent a valid strategy to more classical experimental models for the study of the physiology and pathology of motor neurons preserving the functional anatomy and intrinsic circuitry of the spinal cord.

In this work we used the SNI model to perturb the ventral horn circuitry through the induction of reactive gliosis, focusing the morpho-molecular plastic changes of the tetrapartite synapse. We report that following SNI reactive glial cells surround motor neurons and determine imbalance of synaptic homeostasis and motor neurons dysfunction. Moreover, we found that i.t. administration of GM6001 or the NGF-like peptide BB14 was able to modulate glial activation and the adaptive response of the ECM following PNI, preventing the spinal maladaptive response.

## Materials and Methods

### Animals

We used adult male (250–300 g; Charles River, Calco, Italy) Sprague Dawley rats (n = 60). Animals were allowed free access to food and water, and maintained under a 12/12 h light/dark cycle in pathogen-free iron sheet cages. Experimental procedures and surgery were performed in compliance with the Italian (D.L. 116/92) and European (O.J. of E.C. L358/1 18/12/86) regulation, and approved by the Animal Ethics Committee of the Second University of Naples.

### Spared Nerve Injury model

Spared nerve injury (SNI) of the sciatic nerve was performed according to the methods of Decosterd and Woolf [22]. Rats were anesthetized with tiletamine hydrochloride (30 mg/kg), a skin incision was made on the lateral surface of the thigh, exposing the sciatic nerve and its terminal branches: the sural, common peroneal and tibial nerves. SNI consists in the axotomy and ligation of the tibial and common peroneal nerves, sparing the sural nerve. For the sham-operated control group (SHAM), nerves were just exposed. Every effort was made to avoid stretching or any contact with the sural nerve. We closed muscle and skin in two layers.

### Intrathecal drug delivery

The intrathecal (i.t.) catheter was positioned in the lumbar spinal cord during the surgery to avoid the discomfort bias. A small opening through the lumbar spine vertebral laminas allowed the insertion of the catheter [polyethylene (PE) 10 tubing attached to PE 60 tubing for connection to an osmotic pump] into the subarachnoid space directed to the lumbar spinal cord enlargement. The catheter was safely anchored, the bone breach cemented with a glass ionomer luting cement triple pack (KetacCem radiopaque; 3M ESPE, Seefeld, Germany).

To confirm the correct position of the catheter, lower body paralysis was induced by i.t. lidocaine (2%). Each animal was observed for 2 minutes on a table to evaluate the gait and posture. Only rats demonstrating transient paralysis to the lidocaine injection and lack of motor deficits were used for experimental procedures [infusion of BB14, or GM6001, or artificial CSF (ACSF); n = 15/each group].

After three days, rats were anesthetized by i.t. tiletamine hydrochloride (30 mg/kg) and the free extremity of the catheter was connected to an osmotic minipump [2001 Alzet pumps (Cupertino, CA)] filled with the vehicle (ACSF) containing rat serum albumin (1 mg/ml; Sigma, Italy) and the NGF-like peptide BB14 (37.5 μg/μl), or GM6001 (Calbiochem, Germany) (180 μg/μl), or ACSF only. The pumping rate was of 1 μl/h for 7 days, granting an i.t. infusion dose of 37.5 μg/h (corresponding to 0.9 mg/kg b.w.) of BB14, or 180 μg/h (100 mg/kg b.w.) of GM6001. Osmotic pumps were then implanted subcutaneously.

### Sections preparation

After deep anesthesia with i.t. injection of chloral hydrate (300 mg/kg body weight), animals were transcardially perfused with saline solution (TrisHCl 0.1 M/ EDTA 10 mM), followed by 4% paraformaldehyde/0.1% glutaraldehyde in 0.01 M phosphate-buffer (PBS), pH 7.4 at 4°C. Spinal cords prepared for light microscopy were removed and post-fixed for two hours in the previously mentioned fixative, drenched in 30% sucrose/PBS and then frozen on dry ice in chilled isopentane. Sections of 25 μm of thickness were cut in series with a slide microtome and gathered in cold PBS for immunohistochemistry (IHC).

### Antibodies and IHC of spinal cord

Immunodetection was performed with the following antibodies: goat antibodies to neuronal glutamate transporter EAAC1 (1:4000; Chemicon Temecula, CA, USA); mouse antibodies to Glial Fibrillary Acidic Protein (GFAP) (1:400; Sigma-Aldrich Milano, Italy); guinea pig antibodies directed against glutamate transporter (GLT1) (1:200; Chemicon Temecula, CA, USA); goat antibodies to glycine transporter 1 (GlyT1) (1:1000; Chemicon Temecula, CA, USA); rabbit antibodies recognizing ionized calcium binding adaptor molecule 1 (Iba1) (1:500; Wako Chemicals, VA, USA); rabbit antibodies selective for NGF (1:250; Chemicon Temecula, CA, USA).

Tissue sections were blocked at room temperature (RT) in 10% normal serum in 0.01 M PBS/0.25% Triton-X100 for 1 h. Primary antibodies (GFAP, Iba1, GLT1, GlyT1, EAAC1, NGF) were diluted in 0.01 M PBS containing 10% normal serum and 0.25% Triton. Spinal cord sections were then incubated for 48 hours at 4°C, washed several times in PBS and incubated with the appropriate biotinylated secondary antibody (1:200; Vector Labs Inc., Burlingame, CA, USA) for 90 minutes. After washing in PBS, sections were processed with Vectastain avidin-biotin peroxidase kit (Vector Labs Inc., Burlingame, CA, USA) at RT for 90 min., and then washed in 0.05 M Tris-HCl. The reaction was finalized with 3.3-diaminobenzidine tetrahydrochloride (DAB; Sigma, 0.5 mg/ml in Tris-HCl) and 0.01% hydrogen peroxide. Sections were then mounted on chrome-alume gelatine coated slides, dehydrated and coverslipped.

### High Performance Liquid Chromatography (HPLC) analysis of aminoacids

Reverse Phase-HPLC was performed to analyze aminoacids levels using an Agilent 1200 Series Liquid Chromatograph, with a binary pump delivery system (G1312B), column thermostat (G1316A), robotic autosampler (G1317B) and multi-wavelength detector (G1315B). Borate buffer (0.15 M, pH = 10.2) was used to dilute the sample, with the following addition of the derivatization solution [o-Phthalaldehyde (OPA) (10 mg/ml), β-mercaptoethanol (10 mg/mL)] and diluent solution (mobile-phase A: 1.5% v/v H3PO4).

The mixture (20 μl) was injected after derivatization on a reverse-phase Jupiter 5 μm C18 300 Å (250 mm x 4.6 mm) column at 40°C and the derivatives absorption detected at the wavelength of 338 nm. A flow rate of 1 mL/min with a gradient of the mobile phase A [Na_2_HPO_4_ (10 mM)/Na_2_B4O_7_·10 H_2_O (10 mM)] and phase B [methanol:acetonitrile:water (9:9:2, v:v:v)] were used to obtain the separation. The robotic autosampler automatically derivatized spinal cord samples which were analyzed blindly using amino acid standard samples [glutamic acid, glutamine, glycine and gamma-aminobutyric acid (GABA), 0.25 mM each].

All samples were analyzed for five consecutive times to assess method reproducibility. Concentrations were reported as the Mean of the Peak Areas. Coefficient of variation (% CV) and the standard error of the mean (SEM) were calculated.

Results were expressed as the ratio between the percent of the areas versus the total area of the investigated amino acids.

### Measurements and Statistical analysis

Zeiss Axioskope 2 light microscope with a high-resolution digital camera (C4742-95, Hamamatsu Photonics, Italy) was used to image slides. Markers in the ventral horn of spinal cords (lamina IX) were measured using an image analysis program (MCID 7.0; Imaging Res. Inc, Canada). A morphometric approach was preferred for glial markers due to the precise recognition of single positive elements. Values of GFAP (astrocytic marker) and Iba1 (microglial marker) were expressed as number of positive elements relative to the scanned area (proportional area). The densitometric values of GLT1, GlyT1, EAAC1 and NGF were reported as total target measured area, relative to the scanned area.

Five randomly selected spinal cord sections for each animal were used to obtain the average. The analysis compared treatment groups (SNI + BB14, and SNI + GM6001) versus control groups (SNI + ACSF and SHAM). Raw data were exported and converted using SigmaPlot 10.0 program (SPSS Erkrath Germany) in frequency distribution histograms. All quantitative comparison of data were analyzed by one-way ANOVA [all pairwise Holm-Sidak method for multiple comparisons (*p≤0.01; **p≤0.001)]. All data shown are presented as the mean±SEM.

All the presented images (control and treated groups) were gathered and adjusted for brightness, contrast and sharpness using Adobe Photoshop^®^ Elements software (Adobe Systems Incorporated, San Jose, California, US).

## Results

### MMPs inhibition and neurotrophic support differently modulate astrocytic and microglial response in SNI

Tetrapartite synapse rearrangement following PNI has been shown to be characterized by persistent reactive gliosis and modulation of MMP-mediated neurotrophins expression [13]. In this study, we used the SNI model to gain insight into the role of MMPs and neurotrophic support in modulating motor neurons dysfunction.

**Fig 1:**
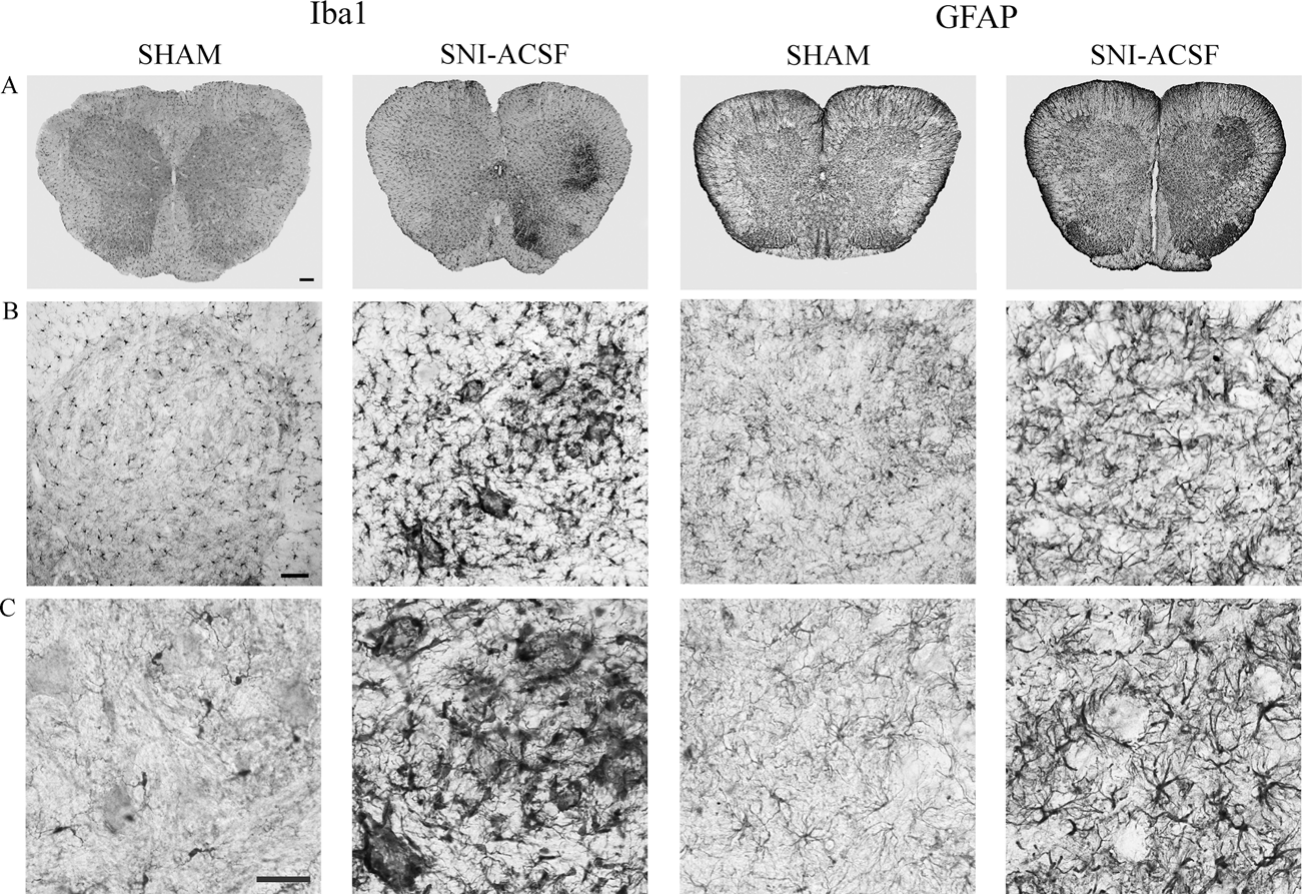
Low (A) and high (B-C) magnification of spinal cord sections stained for glial markers. A) Low magnification (5x) sections show the entire spinal cord in SHAM and SNI rats treated with ACSF. Note, in SNI-ACSF sections, the significant asymmetry of the glial markers expression (indicative of the injured side) and the selective reaction in the dorsal and ventral horn. Scale bar: 200 μm. B) Magnifications (20x) of the ventral horn showing reactive gliosis. Scale bar: 50 μm. C) High magnification (40x) morphological details of reactive microglia and astrocytes. Scale bar: 50 μm.

**Fig 2:**
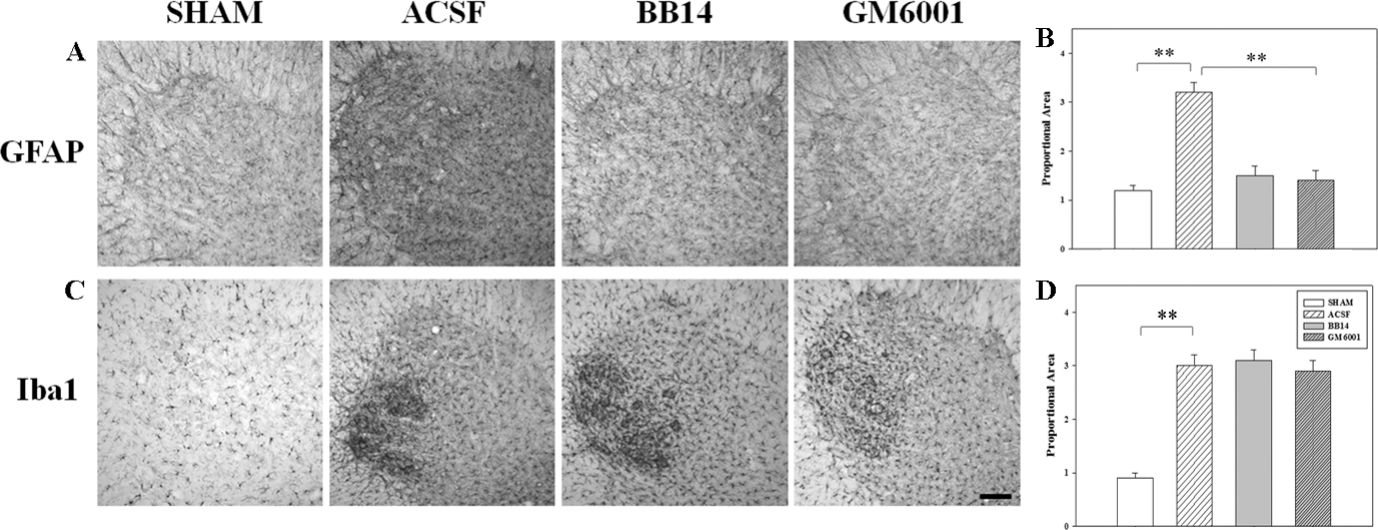
Evaluation of glial markers in the ventral horn of spinal cord. Sections (A and C) and densitometric quantitation (B and D) of ventral horn of lumbar spinal cords from SHAM and SNI-operated animals treated for 7 days with BB14 (0.9 mg/kg b.w.), GM6001 (100 mg/kg. b.w.) or ACSF (vehicle) and immunostained for GFAP (A–B) or Iba1 (C–D). Data are the mean±SEM (**p≤0.001, ACSF vs. SHAM/BB14/GM6001; ANOVA and Holm–Sidak test). Scale bar: 50 μm.

Analysis of glial markers in the lamina IX of the lumbar spinal cord following SNI revealed a sharp increase of GFAP (3.22±0.53) (Figs 1 and 2A and 2B) and Iba1 expression (3.32±0.41) (Figs 1 and 2C and 2D) in the SNI/ACSF-treated animals, as compared to the SHAM group (1.54±0.12 and 1.10±0.05 for GFAP and Iba1, respectively).

**Fig 3:**
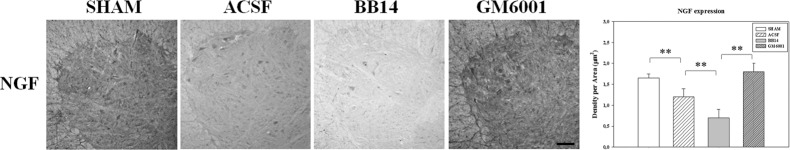
Endogenous NGF expression in the ventral horn. (A) Sections of lumbar spinal cord prepared from SHAM and SNI rats treated for 7 days with ACSF (vehicle), BB14 (0.9 mg/kg b.w.), or GM6001 (100 mg/kg. b.w.) and immunostained with NGF antibody. Scale bar: 50 μm. (B) Densitometric quantitation of NGF levels. Data are the mean±SEM (**p≤0.001, ACSF vs. SHAM/BB14/GM6001; ANOVA and Holm–Sidak test). Scale bar: 50 μm.

To assess the effect of MMPs and neurotrophins in modulating the reactive gliosis, SNI-operated rats were treated with the MMPs inhibitor GM6001. We found that i.t. treatment with GM6001 (100 mg/kg b.w.) for 7 days restored GFAP levels (1.82±0.12) (Fig 2A and 2B), while Iba1 expression was essentially unaffected (2.96±0.25) (Fig 2C and 2D). Moreover, analysis of endogenous NGF levels revealed that NGF expression was significantly decreased in the ventral horn of ACSF-treated animals (1.25±0.22), compared to the SHAM rats (1.60±0.43) (Fig 3) and i.t. administration of GM6001 fully restored the neurotrophin density (1.73±0.13). These data confirm the correlation between the MMPs proteolytic activity in digesting endogenous NGF [13, 22] and the key role of this neurotrophin in modulating astroglial activation.

Based on these results, we further evaluated the efficacy of the NGF-mimetic molecule BB14, which was previously shown to counteract reactive gliosis, and effectively reverse biochemical and behavioral changes induced by SNI in the dorsal horn [22]. I.t. treatment with BB14 (0.9 mg/kg b.w.) for 7 days reduced GFAP levels (1.85±0.16) in the ventral horn of SNI animals, as compared to ACSF rats (Fig 2A and 2B), without affecting Iba1 expression (3.12±0.35) (Fig 2C and 2D) and NGF levels (0.52±0.03) (Fig 3).Thus, both MMPs inhibition by GM6001, which prevented endogenous NGF degradation, and the neurotrophic molecule BB14 reduced the astrocytic reaction in the motor neurons lamina IX, but had no effect on microglia activation. These data, while confirming the role of NGF and BB14 in inhibiting mechanisms of reactive astrocytosis, also indicate that the microglial population has probably different mechanisms of modulation associated with motor neuron damage.

### Modulation of neuronal and glial aminoacid transporters expression in the ventral horn following i.t administration of GM6001 or BB14

Astrocytes play a well-established role in glutamate metabolism and maintenance of synaptic homeostasis [23], instead MMPs have been recently shown to regulate and be regulated by glutamate at excitatory synapses [24–25]. Fine-tuning of excitatory/inhibitory tone is the keystone for synaptic functioning, correct information transmission and neuronal plasticity.

**Fig 4:**
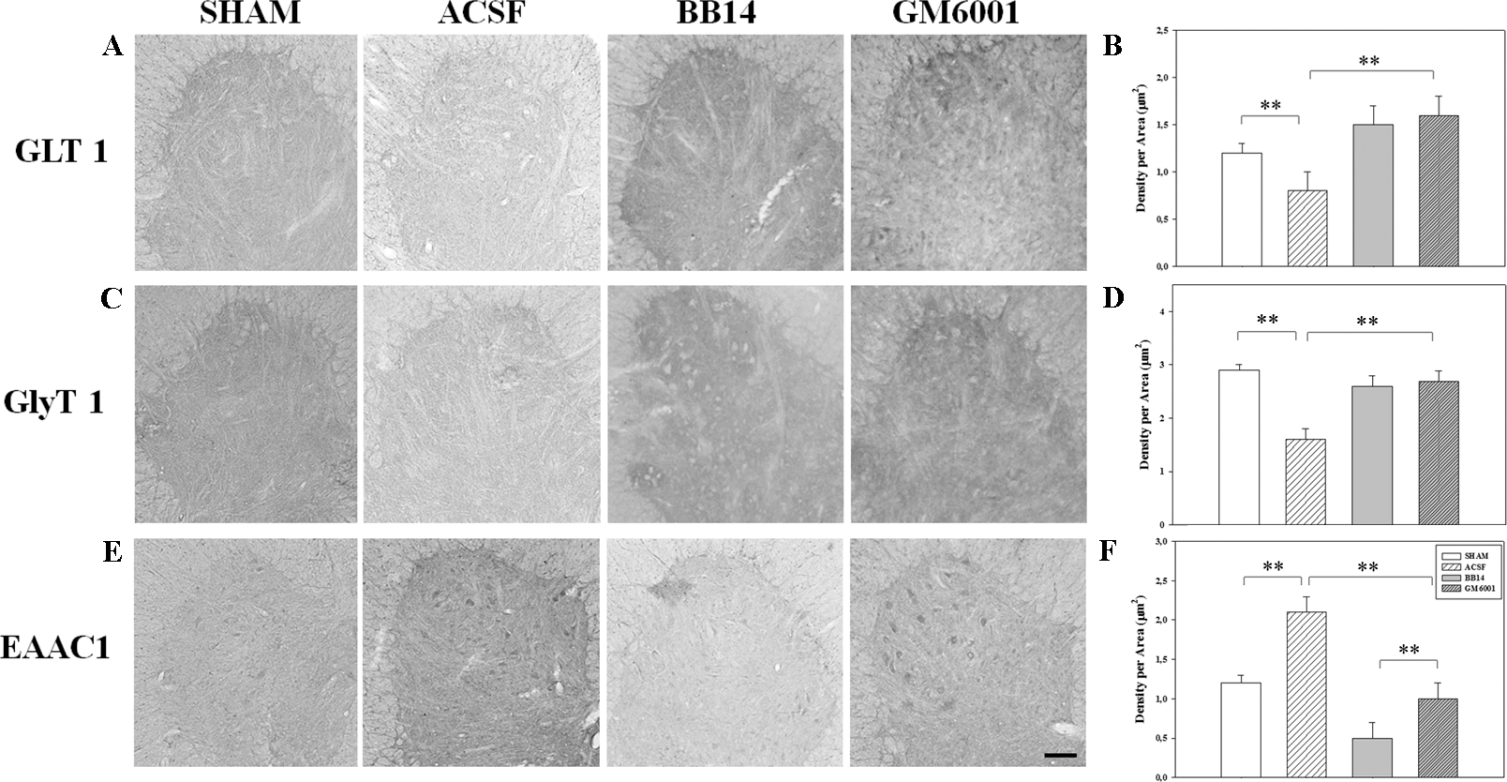
Expression of spinal glial and neuronal aminoacid transporters. Sections of ventral horn of lumbar spinal cord from SHAM and SNI animals treated for 7 days with ACSF (vehicle), BB14 (0.9 mg/kg b.w.), or GM6001 (100 mg/kg. b.w.) and immunostained for glial glutamate (A–B) or glycine (C–D) transporters, or the neuronal glutamate transporter EAAC1 (E–F). Data are the mean±SEM (**p≤0.001, ACSF vs. SHAM/BB14/GM6001; ANOVA and Holm–Sidak test). Scale bar: 50 μm.

Here, we analyzed the connection between reactive gliosis and the alteration of glial glutamate/glycine transporters in the ventral horn of the lumbar spinal cord. IHC analyses of lamina IX displayed a decrease of GLT1 expression in SNI animals (ACSF group) (0.92±0.03), compared to the SHAM group (1.12±0.07) (Fig 4A and 4B). Reduced GLT1 levels in ACSF-treated animals were paralleled by a similar significant decrease of the glycine transporter (GlyT1) expression (1.81±0.42), compared to the levels measured in the SHAM group (2.82±0.31) (Fig 4C and 4D). Both GLT1 and GlyT1 expression were fully recovered by i.t. administration of GM6001 (1.72±0.21 and 2.63±0.32, respectively) or BB14 (1.54±0.22 and 2.52±0.43, respectively).

These data indicate that reactive gliosis and MMPs activation disrupt the homeostasis of synaptic transmission and demonstrate that blocking MMPs activity can significantly restore the expression of the astrocytic transporters, thus preventing mechanisms of maladaptive plasticity. Moreover, the decrease of glial transporters in SNI/ACSF-treated animals was associated with a dramatic increase of the main neuronal glutamate transporter EAAC1, as observed in SNI animals treated with ACSF (2.01±0.43), compared to SHAM rats (1.2±0.20) (Fig 4E and 4F). EAAC1 levels were significantly reduced by i.t. administration of GM6001 (1.12±0.31) or BB14 (0.40±0.03) (Fig 4E and 4F), thus substantiating the key beneficial role of increasing the neurotrophic support on the ventral horn synaptic network, either by modulation of MMPs activity or by BB14 supply.

**Fig 5:**
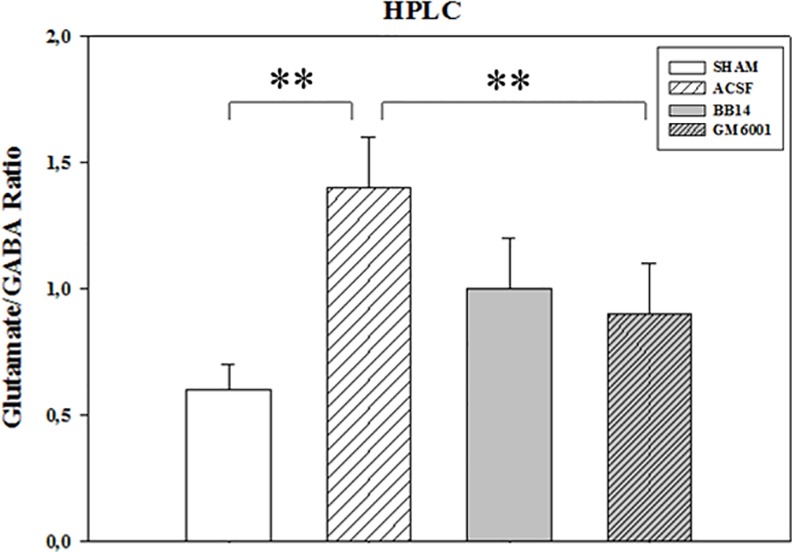
HPLC analysis of Glutamate/GABA ratio. Amino acid levels were measured by HPLC in the ventral horn of lumbar spinal cord dissected from SHAM and SNI animals treated for 7 days with BB14 (0.9 mg/kg b.w.), GM6001 (100 mg/kg. b.w.) or ACSF (vehicle). The Glutamate/GABA ratio was calculated as described in M&M. Data are the mean±SEM (**p≤0.001, ACSF vs. SHAM/BB14/GM6001; ANOVA and Holm–Sidak test).

HPLC analysis (Fig 5) of neurotransmitter levels also demonstrated a significant increase of the glutamate/GABA ratio (1.32±0.31) in SNI animals treated with ACSF compared to the SHAM-operated rats (0.61±0.08). The altered glutamate/GABA balance was partially restored by i.t. GM6001 or BB14 treatment (0.94±0.03 and 0.91±0.02, respectively, *p*≤0.001), further strengthening MMPs and neurotrophins interrelated role in synaptic transmission and response through modulation of glial and neuronal transporters and excitatory/inhibitory balance.

## Discussion

We analyzed ventral horn sections of lumbar spinal cord by IHC following PNI to evaluate morphological and molecular changes in the components of the tetrapartite synapse that characterize the dysfunction of motor neurons [26]. In particular, we focused on the relation between ECM modifiers, such as MMPs, and neurotrophin signaling. Peripheral axotomy produces a serious motor neurons suffering characterized by ECM remodeling, microglia concentric neuropil invasion and reactive astrocytosis (Figs 1 and 2). These changes were accompanied by lowered endogenous NGF levels (Fig 3) and increased glutamatergic tone (Figs 4 and 5). Here we show that both i.t. GM6001 and BB14 administration were able to recover molecular changes in the lamina IX of the spinal cord without influencing microglial response.

Recently our group has proposed the SNI model of the sciatic nerve as a valid tool to perturb the spinal cord homeostasis “from the periphery”, allowing the study of the synaptic remodeling and the morpho-functional changes of motor neurons after peripheral axotomy [21], that leads to progressive wallerian degeneration. Moreover, this model has the advantage to analyze the early changes of tetrapartite synapse and spinal circuitry before motor degeneration occurs. In other words, the inflammasome (as result of microglial and astrocytic activation) overcomes the connectome, thus survival and repair plasticity are warranted at the expense of information processing [27].

MMPs, expressed by neurons, astrocytes and microglia, have a major impact on brain development and synapse function. Recently, it has been shown that an enriched environment could influence MMPs activity and consequently modulates the ECM structure (through MMP-2 or MMP-9 activity) and synapse morphology also in the CNS of adult mice [28]. In addition, the tPA/plasminogen/plasmin/MMP degrading system [12] which breaks down NGF protein, together with TrkA-mediated up-regulation of MMP-9, are part of a variety of connected signaling pathways promoting cell survival, proliferation and MMPs regulation [15].

How mechanisms of reactive gliosis could drive changes in the direction to scar formation, recover of synaptic plasticity or progression to neurodegeneration is the key to understand the balance between adaptive and maladaptive responses. Seven days after peripheral axotomy, we observed clear signs of reactive gliosis (Iba1 and GFAP expression) and a peculiar migration of activated microglial cells through the ECM, sharply surrounding motor neurons cell bodies (Figs 1 and 2), sustaining a neuroinflammatory reaction. Activation of MMPs, moreover, allows cellular migration, being involved in vascular permeability, and promotes cytokine diffusion and activation of proinflammatory cytokines, such as pro-interleukin 1 beta, into the active form. Microglia reactive response, in fact, was found to induce neuronal loss [29] or, in contrast, play a protective role in the CNS. There is currently debate on the modulation of microglial activity [30]. Our data suggests that neither GM6001 nor BB14 were able to prevent or reduce the microglial invasion of the motor neuron neuropil, as expressed by Iba1 levels (Fig 2) and reported in previous studies [31].

These findings demonstrated that pathophysiology of long-term glial response, cornerstone to the establishment of morphological and molecular modifications underlying several neurological diseases [32], is still far to be unraveled.

Spinal glutamate increase represents an excitotoxic stressor and disturbs the stability of synaptic neuroglial network, interfering with synaptic transmission, mechanisms of long term potentiation (LTP), but also neuronal survival [24–25]. The glial glutamate transporters (gGTS) (GLT1, GLAST), expressed by perisynaptic astrocytes, are essential for the reuptake of the glutamate released during synaptic transmission, rescuing the extracellular homeostasis of neurotransmission [33]. Accordingly, the down-regulation of the glial transporters GLT1 and GlyT1 and the compensatory increase of the neuronal glutamate transporter EAAC1 (Fig 4) could represent the direct consequence of glial activation (a calpain-mediated proteolytic process [34]) and a plastic neuronal response to ineffectively quench the overwhelming excitatory glutamate transmission and prevent neuronal death, respectively. This hypothesis is also sustained by our HPLC data, indicating the rise of glutamate/GABA ratio in SNI animals and the significant reduction after GM6001 and BB14 supply (Fig 5).

Glutamate excitotoxicity is currently considered one of the principal mechanisms of the neurodegenerative process and in particular has boost studies on motor neurons death in amyotrophic lateral sclerosis (ALS), a fatal neurodegenerative disorder. In the SOD-1 mice ALS model, in fact, glutamate excitotoxicity was correlated with altered glutamate reuptake function, reduced expression of astrocytic glutamate transporters (GLAST and GLT1) and reduction of neurotrophic factors [35]. The beta lactam antibiotic ceftriaxone was found to increase the expression of GLT1 [36] and was the rationale for a clinical trial with intravenous administration of ceftriaxone in ALS patients. Recently, in immortalized human derived astroglial cells, harmine, a natural beta-carboline alkaloid, was found to activate GLT1 promoter, increase GLT1 gene expression and glutamate uptake activity. In SOD1 mice, harmine effectively increased GLT-1 protein and glutamate transporter activity [37], highlighting the importance of the development of new drugs that could modulate glutamate transporters.

Glial cells are the main source of scar matrix deposition when the CNS-blood barrier is intact [38]. Following injury or disease to the peripheral or CNS, changes in the expression and composition of ECM components can prove detrimental to neural repair [39]. Clustered matrix assemblies as PNNs surround the cell soma, proximal dendrites and axon initial segments of some neurons [40]. The density of PNNs is variable and in the spinal cord PNNs surround approximately 30% of motor neurons in the ventral horn, 50% of large interneurons in the intermediate grey and 20% of neurons in the dorsal horn [41].

Therefore, strategies to manipulate the ECM, such as MMPs inhibitors, could represent a disease-modifying strategy following injury or disease of the brain and spinal cord.

Another important aspect of our data concerns the role of neurotrophins as putative candidate in modulating spinal maladaptive plasticity and restoring the functional synaptic homeostasis and ECM integrity. The neurotrophin system and the ECM remodeling by MMPs are, in fact, substantially connected and influenced by glutamate concentration [24]. In fact, MMPs, strictly regulated, released as inactive pro-enzymes, and constitutively inhibited by the tissue inhibitors of metalloproteinases (TIMPs) [1], cleave all ECM protein components including cell adhesion molecules, receptors and growth factors. In particular, the relevance of neurotrophic support on morphological and biochemical changes linked to reactive gliosis was confirmed by the reduction of endogenous NGF levels following SNI (Fig 3), which were restored by i.t. GM6001 administration [12], thus confirming a primary role of MMPs in degrading this neurotrophin. The importance of ECM in maintaining and promoting neural homeostasis is highlighted by a recent study in which the synthetic tetracycline minocycline, endowed with a non-selective, weak MMP inhibitory activity, was found to promote functional recovery after spinal cord injury in a phase II clinical trial [42].

In conclusion, modulation of the glial reaction, ECM components degradation and neurotrophic factors may be fundamental to spinal motor neuron vitality and synaptic homeostasis following PNI, suggesting that new therapeutic strategies should focus on the four components of the synapse, drawing the unknown paths that connect all the factors responsible for maladaptive plasticity.

## Conclusion

MMPs may induce tissue injury by direct neurotoxic effects on the tetrapartite synapse or interfering with the neurotrophic balance.

Our findings focused the phenotypic microglial changes and astrocytic reaction in determining maladaptive plasticity in the lamina IX of the spinal cord following PNI. The reduction of gGTs and the increase of EAAC1 perturbed the synaptic circuitry balance reducing the glutamate and glycine uptake, with following outflow of neurotransmitters in the extra-synaptic space, and the glutamate/GABA ratio changes. Neuroprotection by endogenous NGF was lowered and microglia activation surrounded ventral horn neuronal cells.

On the other hand, our data strongly support the role played by GM6001 and BB14 in modulating glial function and restoring some aspects of synaptic homeostasis by rescuing the glutamatergic components and their control of synaptic glutamate levels, restoration of endogenous NGF expression without modification of microglial components.

Although mechanisms of MMPs activity in neuroinflammatory or degenerative diseases remain unclear, we found strong evidence that MMPs contribute to maladaptive plasticity in the ventral horn of the spinal cord following PNI.

Future studies clarifying the role of MMPs and their tissue inhibitors as potential therapeutic targets will open new avenues for a better understanding of CNS structure and function. In a longer perspective, this may also provide the opportunity for curative intervention, preventing the connectome reactive miswiring.
